# Development of Thermally Stable Nanobodies for Detection and Neutralization of Staphylococcal Enterotoxin B

**DOI:** 10.3390/toxins15060400

**Published:** 2023-06-16

**Authors:** Anna C. Hughes, Marina Kirkland, Wenxian Du, Reuven Rasooly, Bradley Hernlem, Christina Tam, Yuzhu Zhang, Xiaohua He

**Affiliations:** Western Regional Research Center United States Department of Agriculture, Agricultural Research Service, 800 Buchanan St., Albany, CA 94710, USA; annacarolhughes@gmail.com (A.C.H.); Me.kirkland.mitchell@gmail.com (M.K.); wen-xian.du@usda.gov (W.D.); reuven.rasooly@usda.gov (R.R.); bradley.hernlem@usda.gov (B.H.); christina.tam@usda.gov (C.T.); yuzhu.zhang@usda.gov (Y.Z.)

**Keywords:** camelid single-domain antibody, bivalent nanobody, enzyme-linked immunosorbent assay, limit of detection, milk, staphylococcal enterotoxin, thermal stability

## Abstract

In this study, sixteen unique staphylococcal enterotoxin B (SEB)-reactive nanobodies (nbs), including ten monovalent and six bivalent nbs, were developed. All characterized nbs were highly specific for SEB and did not cross-react with other staphylococcal enterotoxins (SE). Several formats of highly sensitive enzyme-linked immunosorbent assays (ELISAs) were established using SEB nbs and a polyclonal antibody (pAb). The lowest limit of detection (LOD) reached 50 pg/mL in PBS. When applied to an ELISA to detect SEB-spiked milk (a commonly contaminated foodstuff), a LOD as low as 190 pg/mL was obtained. The sensitivity of ELISA was found to increase concurrently with the valency of nbs used in the assay. In addition, a wide range of thermal tolerance was observed among the sixteen nbs, with a subset of nbs, SEB-5, SEB-9, and SEB-6^2^, retaining activity even after exposure to 95 °C for 10 min, whereas the conventional monoclonal and polyclonal antibodies exhibited heat-labile properties. Several nbs demonstrated a long shelf-life, with one nb (SEB-9) retaining 93% of its activity after two weeks of storage at room temperature. In addition to their usage in toxin detection, eleven out of fifteen nbs were capable of neutralizing SEB’s super-antigenic activity, demonstrated by their inhibition on IL-2 expression in an ex vivo human PBMC assay. Compared to monoclonal and polyclonal antibodies, the nbs are relatively small, thermally stable, and easy to produce, making them useful in applications for sensitive, specific, and cost-effective detection and management of SEB contamination in food products.

## 1. Introduction

*Staphylococcus aureus* (*S. aureus*) is a common opportunistic pathogen that colonizes human skin as well as domestic animals. Approximately 50% of people are carriers (persistent or intermittent) of *S. aureus*, which can lead to inadvertent contamination during food preparation [1,2,3,4,5]. Consequently, over 200,000 foodborne illnesses in the US result from *S. aureus* each year [6]. Complications resulting from staphylococcal food poisoning (SFP) are largely caused by toxicity of the super-antigen (SA) staphylococcal enterotoxin B (SEB) [7]. Super-antigens subvert the host immune system by nonspecifically crosslinking T-cell receptors to the major histocompatibility complex class II (MHC II) molecules on antigen-presenting cells (APCs) [8,9,10,11]. Up to 20% of T-cells are stimulated by SAs (compared to 0.001% of the conventional peptide-specific T-cell response) [11,12,13,14]. The resulting unregulated release of proinflammatory cytokines is characteristic of toxic shock and can be lethal [14,15]. Moreover, SEB is resistant to proteolytic digestion, heat treatment, and low pH, allowing it to remain active in environments, such as the digestive tract or processed foods, where even the organism that produces the toxin cannot persist [16,17,18]. Consumption of as little as 20 ng/kg of enterotoxin rapidly results in abdominal cramps, nausea, vomiting, and sometimes diarrhea [19,20,21]. Due to its high stability, toxicity, and ease of dissemination, SEB has been classified as a class B priority toxin (https://www.niaid.nih.gov/research/emerging-infectious-diseases-pathogens, accessed on 1 June 2023) and is considered a potential bioterrorism agent [22]. 

Due to the classification of SEB as a bioterrorism agent and its propensity to cause SFP, there is a need for continued improvement of diagnostic and surveillance strategies against SEB. Conventional monoclonal (mAb) and polyclonal antibodies (pAb) have been shown to be effective at neutralizing the immune response to SEB [23] as well as protecting against a lethal dose of SEB in vivo [24]. There are also a myriad of immunoassays using mAb for the detection of SEB [25,26,27]. However, mAbs are laborious to screen, costly to produce, and limited in applications due to their heat-labile nature, while pAbs often lack specificity in antigen binding. As an alternative to conventional antibodies, single-domain antibodies (sdAb) are a versatile tool compatible with most immunoassay formats and are being developed for an array of promising therapeutics [28].

A nanobody (nb) is a recombinant sdAb derived from the variable region of the heavy-chain-only antibody (VHH) found in camelids [29] or sharks [30]. It is small (~15 kDa), highly soluble [31], and thermally stable [32,33] when compared to traditional antibodies. Moreover, the ease of recombinant expression and multimerization [34,35] of nbs make for facile customization of a single functional molecule with multiple binding properties. 

Previous studies have demonstrated the effectiveness of isolating SEB nbs and applying them in different immunoassays for the detection of SEB. However, most of these studies only focused on the development and characterization of a single or very few nbs [36,37,38,39]. Here, we report the isolation and characterization of 16 unique SEB-reactive nbs (10 monovalent and 6 bivalent) and their physical and chemical properties, including their binding affinity and specificity to SEB in ELISAs, thermostability, shelf-life, and capacity to neutralize the immune response to SEB. The information will help readers to gain a comprehensive understanding of nbs and their potential use in different applications.

## 2. Results

### 2.1. Identification of SEB-Reactive Nanobodies

**Figure 1:**
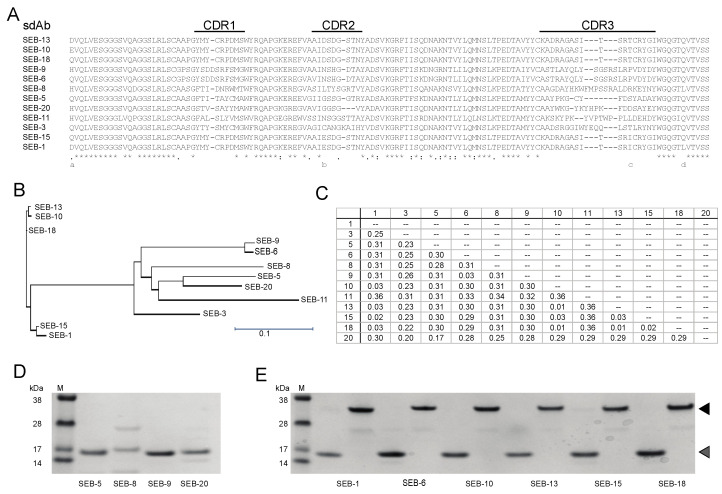
Identification and purification of unique SEB nbs. (**A**) Sequence alignment of nbs. Sequence alignment was generated using Clustal Omega. An asterisk (*) indicates positions with conserved residues, a period (.) indicates conservation between groups of weakly similar properties, and a colon (:) indicates conservation between groups of strongly similar properties. CDR’s are indicated above the alignment. (**B**) Phylogenetic tree of unique nbs. A neighbor-joining tree was generated using the Clustal Omega aligning feature in Lasergene MegAlign Pro software from DNASTAR, Inc. Scale bar represents the branch length. (**C**) Distance matrix table. The matrix shows the uncorrected pairwise distance with global gap removal. (**D**,**E**) Analysis of purified SEB nbs. Panel (**D**) shows Coomassie-stained SDS-PAGE with 0.5 µg of SEB nbs that were only soluble in monomeric form. Panel (**E**) shows Coomassie-stained SDS-PAGE with 0.5 µg of SEB nbs that were soluble in both monomeric and dimeric forms. The molecular weights (kDa) of protein markers (M) are indicated next to the markers. Expected monomeric and dimeric sizes are indicated by the grey and black arrows, respectively.

To generate nanobodies against SEB, one camelid was immunized with a SEB toxoid as the antigen. Total RNA was isolated from PBMCs of the immunized camelid and the coding sequences of VHH were amplified by RT-PCR for construction of an enriched SEB-binding phage display library. Upon transformation into *E. coli* strain TG1, a VHH phagemid library was constructed with a diversity of 1.7 × 10^9^. Phage antibody particles displaying the VHH were produced and subjected to three cycles of bio-panning in plates coated with SEB. Forty clones were randomly picked from the 3rd round-enriched pool to validate the specificity of enrichment. Through DNA sequencing, 12 unique VHH sequences were identified and a neighbor-joining tree revealed clusters of similar sequences (Figure 1A–C). There were several highly similar VHHs that only differed in a few amino acids in framework or CDR regions, such as VHHs SEB-1, SEB-15, SEB-10, SEB-13, and SEB-18. However, highly divergent VHHs were also identified. For example, VHHs SEB-6 and SEB-9 are 98% identical but share at most 75% sequence identity with the other ten nbs. VHHs SEB-5 and SEB-20 shared the least sequence similarity to the other 10 nbs. We suspected that differences in the framework sequences would result in heterogeneity of protein stability or solubility, whereas CDR sequence variability might affect SEB reactivity. Therefore, we set out to further purify and characterize these VHH nbs.

### 2.2. Production and Characterization of SEB nbs and pAb

To further characterize the SEB nbs, each nb sequence was cloned into a LIC vector (https://www.addgene.org/29653/, accessed on 1 June 2023) and expressed in *E. coli* cells, and the nb was purified. Three out of twelve nbs were insoluble (SEB-3 and SEB-11) or poorly expressed (SEB-8) and were therefore excluded from further study. Among the 9 remaining nbs, 7 were soluble and well-expressed, yielding between 10 and 78 mg of protein from a 1 L *E. coli* culture, which were significantly higher than the nbs yields (5–20 mg/L) reported previously [37,38], and 2 (SEB-5 and SEB-9) were partially soluble and when purified, resulted in a yield of only 0.32 mg and 6.8 mg of protein from a 1 L *E. coli* culture, respectively (Table 1). Furthermore, SEB-5 required a second purification step to produce a protein with a single distinct band at the correct size of 15 kDa on a Coomassie-stained SDS-PAGE gel (Figure 1D,E). 

**Figure 2:**
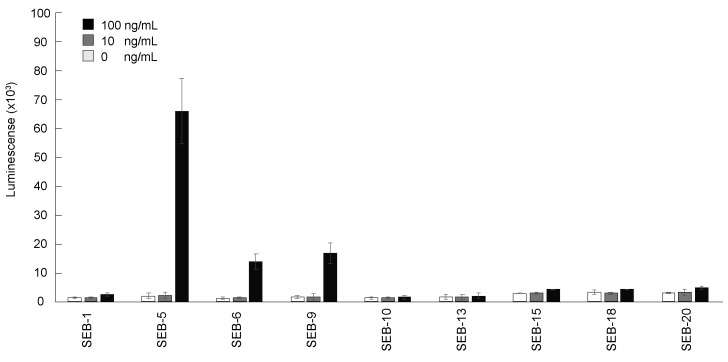
Detection of SEB by direct ELISA using nbs. The 96-well plates were coated with SEB 0 ng/mL (white bars), 10 ng/mL (grey bars), and 100 ng/mL (black bars), and then incubated with each nb conjugated with biotin (200 ng/mL), and then with streptavidin-HRP before ELISA signal development with the SuperSignal West Pico Chemiluminescent substrate. Bars represent the mean of triplicate readings ± one standard deviation from a representative experiment. Each experiment was repeated 3 times.

The purified nbs were then tested for their reactivity to SEB by direct ELISA. Figure 2 shows that the nine nbs bound SEB in different capacities, with nb SEB-5 producing the highest signal. However, the sensitivity of detection was poor, with the limit of detection (LOD) being above 10 ng/mL for all nbs tested by direct ELISA. This result was similar to the results reported previously [36].

One way to increase the sensitivity of detection without a loss of specificity is to utilize sandwich ELISAs [40]. A sandwich ELISA measures the amount of antigen between two layers of antibodies (capture and detection antibodies). The antigen to be measured must contain at least two antigenic sites capable of binding to antibodies, since at least two antibodies act in the sandwich. Therefore, a new pAb was developed using the SEB toxoid as the antigen. The two rabbits immunized with the toxoid showed high serum antibody titers (≥1:8000) in ELISA using plates coated with 10 ng/mL of SEB (Figure S1A). Western blot analysis showed that the pAb IgG detected the SEB active toxin (Figure S1B). Each nb was then re-evaluated by pairing with the pAb in sandwich ELISAs. As expected, pairing nbs with the pAb in a sandwich ELISA remarkably increased the sensitivity of detection. Table 1 indicates that the best ELISAs were obtained by using the pAb as a capturer and SEB-5 or SEB-15 nbs as a detector. The LOD from these two ELISAs was 190 pg/mL, much lower than the ELISA results obtained from direct ELISAs. Compared with the LOD obtained from a similar assay format that used a mAb as a capturer and a nb-alkaline phosphatase fusion protein as a detector [36], this LOD was about 7.5-fold lower. To avoid the need of biotinylating the detection antibodies, we also used the nbs as capture antibodies and the pAb as the detecting antibody. The best antibody pair in this setting was SEB-5/pAb, with a LOD of 390 pg/mL (Table 1).

**Figure 3:**
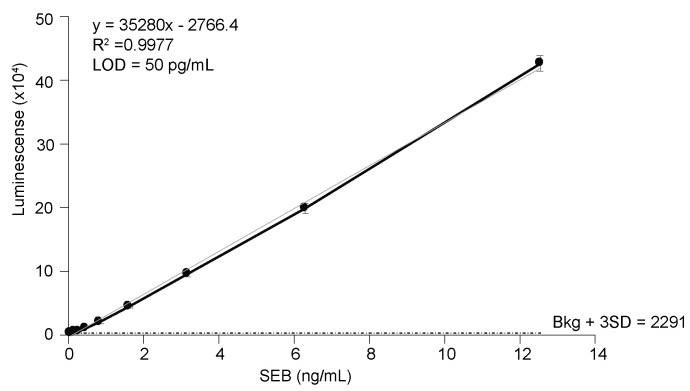
Detection of SEB in PBS by sandwich ELISA. SEB polyclonal antibody (1 ug/mL) was used as a capture antibody and biotinylated nb SEB-1^2^ (0.2 µg/mL) was used as a detection antibody. Black circles represent the mean of three replicates ± one standard deviation. The horizontal dashed line equals the mean counts from PBS (no toxin control) plus three SD.

It has been reported that the binding affinity of a nb for its substrate/antigen can be easily increased through oligomerization without compromising the intrinsic properties of the nb [34,41,42]. Therefore, bivalent nbs were generated by expressing tandem VHH sequences connected by a flexible serine-glycine linker sequence (bivalent forms of nb are indicated by either SEB-#^2^ or are explicitly stated to be bivalent). The yields of generated bivalent nbs are indicated in Table 1. Bivalent nbs SEB-5^2^, SEB-9^2^, and SEB-20^2^ were insoluble. It is notable that the corresponding monovalent forms of these three insoluble bivalent nbs had poor solubility and low protein yields. However, the remaining 6 nbs in bivalent form were successfully expressed as 30 kDa soluble proteins (Figure 1E). As previously reported [34,41,42], the bivalent forms of the nbs had a stronger binding affinity than their monovalent forms in most cases (Table 1). It was found that the sandwich ELISA using SEB pAb as a capturer and the SEB-1 bivalent nb as a detector exhibited the best sensitivity, with a LOD of 50 pg/mL (Figure 3). This is the most sensitive ELISA for SEB, to our knowledge. 

Nbs with sequence variations in CDR may suggest that these nbs bind to distinct SEB epitopes and thus might serve as compatible antibody pairs in a sandwich ELISA. To identify the best nb pair for a sandwich ELISA, all possible combinations of nb pairs were evaluated using each of the nbs, with significant variations in CDR as either the capture or the detector antibody. We found that the best result was obtained when using nb SEB-1^2^ as a capture antibody and biotinylated nb SEB-20 as a detector. This ELISA detected as little as 780 pg/mL of SEB. The five most sensitive nb pairs are shown in Table 2. Based on the ELISA data, we inferred that SEB-1 and SEB-9 bind distinct epitopes from either SEB-5 or SEB-20. Further, there was no signal from SEB-1 when paired with SEB-9 or when SEB-5 was paired with SEB-20 (data not shown). It is possible that these pairs bind to overlapping epitopes or in some way occlude binding of the second nb. 

### 2.3. Heat Stability

**Figure 4:**
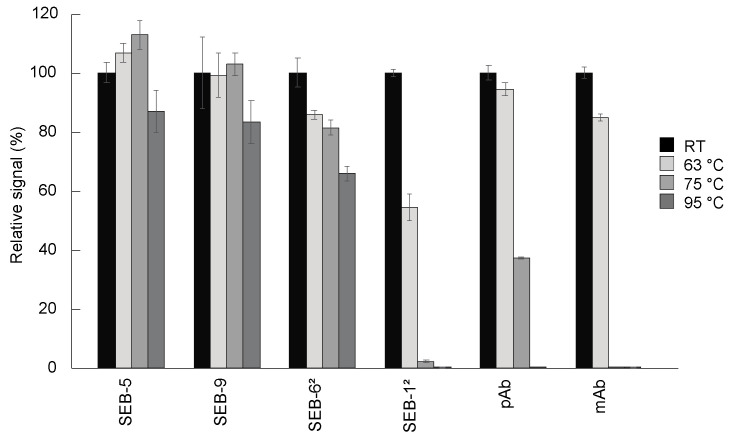
Thermostability of nbs. Antibodies including nbs, mAb, and pAb were diluted to 100 µg/mL in PBS and then incubated for 10 min at room temperature (black bars), 63 °C (light grey bars), 75 °C (medium dark grey bars), and 95 °C (dark grey bars), respectively. These antibodies were then diluted to 200 ng/mL for use in a direct ELISA. For the direct ELISA, plates were coated with 5 µg/mL of SEB, and then incubated with antibodies. After adding appropriate secondary antibodies, the plates were developed with 100 µL of substrate for signal generation. The ELISA signal was corrected by subtracting the average of the negative control (PBS) and then scaled to the room temperature-treated antibodies. Bars represent the mean of triplicate readings ± one standard deviation from one representative experiment. Each experiment was repeated 2 times.

One of the hallmarks of camelid-derived nbs is their ability to reversibly melt and refold across a broad range of temperatures that are not permissible with conventional antibodies [32,33]. We assessed the heat stability of the SEB nbs by measuring their activities with a direct ELISA after incubation for 10 min at 63 °C, 75 °C, or 95 °C, compared to antibodies held at room temperature (Figure 4). The most stable molecules were SEB-5 and SEB-9, which retained more than 80% of the binding activity, followed by the SEB-6^2^ nb, which retained more than 60% of the binding activity after 10 min at 95 °C. The next most stable antibody tested was the pAb, which was partially active after treatment at 75 °C, but completely inactivated after treatment at 95 °C for 10 min. Finally, SEB-1^2^ and the mAb were only partially active after treatment at 63 °C, and almost completely inactivated at 75 °C and above. Previous studies [33] showed that an SEB nb retained 100% of its binding activity after 20 min at 95 °C, while a conventional antibody lost all binding after 10 min at 95 °C. These data suggest that most nbs are more thermally stable than conventional antibodies and could be preferable options in assays designed to minimize the effects of temperature fluctuations. 

### 2.4. Shelf-Life

**Figure 5:**
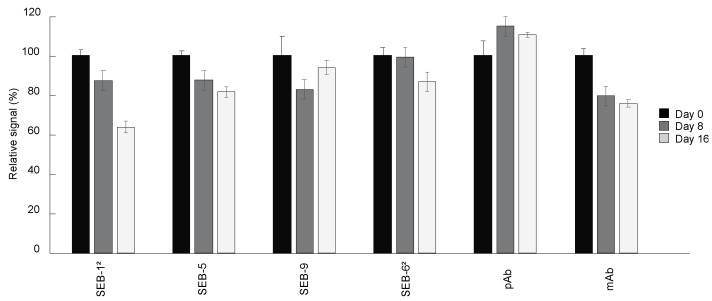
Shelf-life of nbs. Antibodies including nbs, mAb, and pAb were stored at room temperature for 0 (black bars), 8 (dark grey bars), and 16 (light grey bars) days, respectively. Direct ELISA was performed by coating plates with 5 µg/mL of SEB, and then incubated with antibodies stored at room temperature for different time lengths, followed by adding secondary antibodies and signal development. The ELISA signal was corrected by subtracting the average of the negative control (PBS) and then scaled to the antibodies stored at 4 °C (0 day at room temperature). Bars represent the mean of triplicate readings ± one standard deviation from one representative experiment. Each experiment was repeated 2 times.

The shelf-life of immunoassay diagnostic tools is largely determined by the antibody stability under certain storage conditions. To determine the suitability of the SEB nbs for use in an ELISA kit stored at room temperature, changes in the binding activity of SEB nbs stored at room temperature relative to nbs stored at 4 °C were measured. Unsurprisingly, there was no significant loss of signal by the pAb stored for two weeks at room temperature compared with that stored at 4 °C. In contrast, the signal of the mAb, SEB-1^2^, and SEB-5 decreased by 25%, 36%, and 19%, respectively, over a two-week period. The nbs with the longest shelf-life, SEB-9 and SEB-6^2^, maintained 93% and 86% of binding activity after storage at room temperature for two weeks (Figure 5). 

### 2.5. Specificity of SEB nbs

As many as 29 staphylococcal super-antigens have been identified [43]. Among them, SEA, SEB, SEC, SED, and SEE are the five major SEs commonly associated with SFP. These SAs are highly similar in structure [44]. To test if they are immunologically distinct, we investigated the cross-reactivity of the nbs to SEA, SEE, and SED (note: SEC was not tested because it was not available during this study). All the nbs developed in this study, as well as the pAb, could not detect 100 ng/mL of SEA, SEE, or SED when tested in a direct ELISA (Figure S2). We conclude that the nbs and pAb are highly specific for SEB.

### 2.6. Detection of SEB in Milk

*S. aureus* is capable of growing and producing toxins in dairy products, which if consumed could result in illness in as few as three hours [5,19,45]. Therefore, we wanted to determine if the nbs developed here can be used in assays for the detection of SEB in milk. The four most sensitive capture nbs were tested in a sandwich ELISA, paired with the pAb. No matrix effect was found in toxin-spiked milk (whole and 2%) compared to PBS. The most sensitive ELISA for SEB in milk was using the antibody pair SEB-1^2^/pAb (Table 3), which had a LOD between 190 and 390 pg/mL. Nb-based immunoassays with similar sensitivity for the detection of SEB in milk were also previously reported [36,37,39]. We conclude that these SEB-reactive nbs could be reliably applied for use with dairy products. 

### 2.7. Nanobody Neutralization of SEB

**Figure 6:**
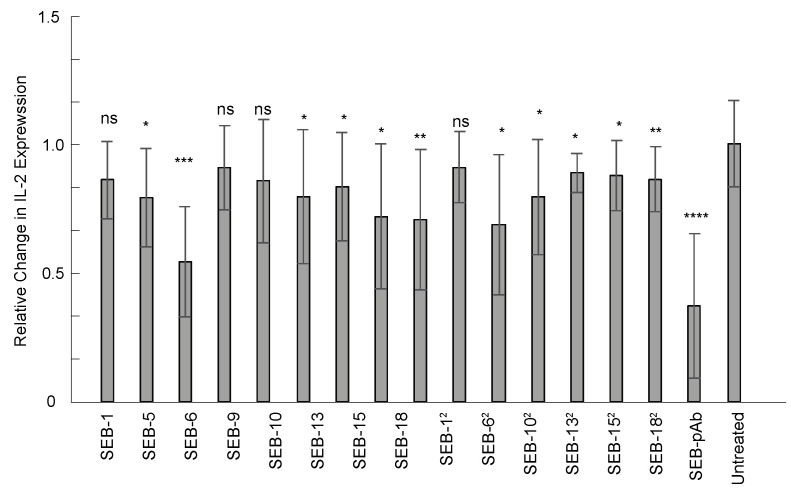
Nanobody neutralization of SEB toxin ex vivo. Human PBMCs were co-incubated with 25 ng/mL of SEB or 25 ng/mL of SEB with an equal concentration of nanobody or anti-SEB polyclonal antibody for 24 h at 37 °C. IL-2 expression was measured from the culture supernatant using an ELISA and normalized to the group of PBMCs treated with SEB (without antibodies). Results display combined data from three independent experiments, n = 9. Statistics used were the Mann–Whitney test with SEM. ns indicates *p* > 0.05, statistically insignificant; *, **, ***, and **** indicate statistically significant at *p* ≤ 0.05, *p* ≤ 0.01, *p* ≤ 0.001, and *p* ≤ 0.0001, respectively.

As a super-antigen, SEB is a potent activator of the immune system, capable of promoting the unregulated release of proinflammatory cytokines that can result in toxic shock. Currently, there are no approved therapies for SEB. Passive immunotherapy with engineered nbs could be an option for SEB exposure. Therefore, the anti-SEB nanobody panel was screened for neutralization properties in an ex vivo human PBMC assay. IL-2 expression was measured using an ELISA (Biolegend) after PBMCs were co-incubated with SEB alone, SEB with anti-SEB pAb, or SEB with nanobodies for 24 h. Of the 15 nbs screened, 11 exhibited significant neutralization of SEB compared to the control group without antibody treatment (*p* ≤ 0.05), but the greatest reductions in IL-2 expression were with SEB-6, SEB-20, SEB-18, and SEB-6^2^ (Figure 6). These data demonstrate that the nanobodies have neutralization properties and that their applications can extend beyond detection assays.

## 3. Discussion

SEB is one of the most potent super-antigens responsible for SFP and is an NIH class B priority toxin [22]. The low toxic dose of SEB and its potential use as a bioterrorism agent necessitate sensitive tools for detection and neutralization [19,20,21]. Several nbs and nb-based immunoassays for detection of SEB in buffer and milk have been reported, with varying degrees of sensitivity and specificity [36,37,38,39]. However, the diverse properties of nbs were not well-reflected in those studies because very few nbs were characterized. In this study, we identified 16 SEB-reactive nbs and assessed their utility in various applications under multiple conditions. We found that all the nbs screened here varied in solubility, sequence, and binding affinity to SEB when tested in ELISAs, as well as thermal and temporal stability and neutralization capability. Although not all nbs may be capable of competing with conventional mAbs and pAbs in terms of detection sensitivity and neutralization capability, they had advantages such as small size, stability in harsh conditions, low production costs, and their potential to be engineered to fit the needs of the final applications.

In this study, several nbs were highly similar. In fact, SEB-10, SEB-13, and SEB-18 were identical in their CDR sequence and only differed by one amino acid (AA) in the framework. All three were soluble and stable in a bivalent form but were poor detectors for SEB toxin. In contrast, SEB-1 and SEB-15, which shared 97% sequence identity with the SEB-10/13/18 cluster, were among the most sensitive bivalent nbs when tested by ELISA. The two AA differences between the SEB-10/13/18 cluster and the SEB-1/15 cluster, one in CDR2 and one in CDR3, are likely responsible for the variation in the toxin reactivity of the two groups. Of all the nbs, SEB-1^2^ produced the lowest LOD when paired with either a capturing pAb or a detecting SEB-20 nb, so it might be a good candidate for further development in sandwich ELISAs.

Reagent shelf-life is an issue for biosensors. As such, nbs are ideally suited for use in immuno-biosensors due to their stability and long shelf-life. The nbs developed here demonstrated a range of stability over a two-week period when stored at room temperature. SEB-1^2^ was one of the more sensitive nbs in the ELISA, however, it was the least stable over time. In contrast, SEB-6^2^ and SEB-9 maintained 86% and 93% activity, respectively, after long-term storage. SEB-6^2^ or SEB-9 might be suitable tools for use in detection platforms, such as lateral flow strips that are commonly packaged and stored at ambient temperatures. 

*S. aureus* is a common dairy contaminant, and environments encountered during food production and processing can result in fluctuations in SEB production [17]. Therefore, it is important for tools used in food safety to be able to function in nonstandard lab conditions. When challenged to temperatures commonly used during pasteurization, several of the SEB nbs demonstrated high thermal tolerance. Indeed, SEB-5, SEB-9, and SEB-6^2^ maintained partial functionality after exposure to 95 °C, with SEB-5 retaining over 80% activity. With the high degree of thermal stability combined with an activity that persists in the presence of dairy products, SEB-5 is a good candidate for further development as a reagent used in dairy production. 

In summary, a suite of SEB-reactive nbs were developed. Each had its own unique characteristics, with some suited to applications that require tolerance to high temperatures, or high sensitivity for diagnostics, whereas others were more suited for therapeutics or assays requiring a long shelf-life. One of the advantages of using nbs instead of conventional antibodies is the ease at which they can be genetically manipulated. We generated bivalent nbs by utilizing a flexible linker between two monovalent nbs and found that in most cases, the bivalent nbs outperformed the monovalent nbs when they were tested in ELISAs. According to previous reports, it is possible to increase the valency well-above two through genetic approaches [41,46]. As such, the nbs developed here could be used to generate a monospecific multimer, exhibiting multiple functions with respect to the SEB reactivity. For example, SEB-6^2^ linked with SEB-1^2^ could generate a molecule that can both detect and neutralize SEB. Further, the SEB nbs could be combined with heterospecific nbs to generate a multimeric protein that can target multiple foodborne toxins simultaneously. 

## 4. Materials and Methods

### 4.1. Immunization of Camelid and Antibody Phage Display Library Screening

SEB-reactive nbs were screened by phage display technology using a library developed from an immunized camel (Creative Biolabs, Ramsey Road, Shirley, NY, USA). A SEB toxoid (List Biological, Campbell, CA, USA LIST Labs) was used as an antigen for subcutaneous immunization of one camelid (*Camelus bactrianus*) and the subsequent generation of a SEB nb phage display library. Briefly, the camel was immunized five times with a three-week interval between injections of an emulsion of the mixed toxoids, with an equal volume of complete Freund adjuvant (600 μg/600 μL). Peripheral blood mononuclear cells (PBMCs) from 400 mL of blood were isolated by centrifugation with Ficoll Hypaque density gradient centrifugation. Total RNA was isolated from PBMCs using TRIzol reagent and used for RT-PCR of the nb sequences. The nb coding sequences were then ligated into pDISPLAY-3MTm and used to generate the nb phage display library. SEB-binding nbs were enriched by four rounds of bio-panning against the SEB toxoid, and unique nbs were identified by DNA sequencing. 

### 4.2. Bioinformatics

The unique nb sequences were aligned using Clustal Omega [47]. Lasergene MegAlign Pro software (DNASTAR, Inc, Madison, WI, USA) was used to generate a neighbor-joining tree and a distance matrix table, showing the uncorrected pairwise distance with global gap removal. 

### 4.3. nb Cloning, Expression, and Purification

Monovalent nb DNA fragments with a LIC fusion tag (5′TACTTCCAATCCAATGCA3′ at the N-terminus and 5′TTATCCACTTCCAATGTTATTA3′ at the C-terminus) were synthesized by IDT (Coralville, IA, USA). The synthesized DNA was then cloned into the pET His6 TEVLIC vector-1B following the instructions for the Addgene plasmid #29653 (https://www.addgene.org/29653/, accessed on 1 June 2023). The ligated plasmid was verified by PCR and transformed into either BL21 (ThermoFisher, Emeryville, CA) or T7 Shuffle cells (New England Biolabs, Ipswich, MA) for protein expression.

Bivalent nb fragments were produced by overlapping PCR. Primers (Table S1) encoding the sequence of a serine glycine linker (GGGSGGGSGGGS) on either the 5′ or 3′ end were used to amplify DNA from the synthesized DNA from IDT. The resulting fragments were then gel-extracted for purity, mixed in equal molar ratios, and used as the template for a second round of PCR using the LIC primers. The resulting cemented amplicon was then cloned into the pET His6 TEVLIC vector-1B, as described above, and transformed into Shuffle T7 cells.

For monovalent and bivalent nb expression, an overnight culture (20 mL) was diluted 1:50 into 1 L of Lysogeny Broth (LB) medium supplemented with 50 μg/mL of kanamycin. The cell cultures were then incubated with shaking at 37 °C (BL21) or 30 °C (T7 Shuffle) until reaching an optical density of 0.6 at the wavelength 600 nm (OD600); at this point, protein production was induced by the addition of isopropylβ-d-1-thiogalactopyranoside (IPTG) at a final concentration of 1 mM at 16 °C for 20 h (BL21) or 30 °C for 3 h (T7 Shuffle). The cells were harvested by centrifugation at 8000× *g* for 10 min at 4 °C. The cell pellet was lysed in a 1:10 volume of nickel NTA buffer (20 mM Tris pH 8, 300 mM NaCl, 20 mM Imidazole, 10% Glycerol). Lysates were clarified by centrifugation at 15,000× *g* for 10 min at 4 °C and purified by affinity chromatography using a nickel NTA column HisTrap™ Fast Flow (Sigma-Aldrich, Inc. St. Louis, MO, USA) with elution buffer (20 mM Tris pH 8, 300 mM NaCl, 10% glycerol, 500 mM imidazol). The purified protein was concentrated, and buffer-exchanged into 1× phosphate-buffered saline (PBS), and when needed, biotinylated using the Lightning-Link^®^ Rapid Type A Biotin Antibody Labeling Kit (Novus biologics, Centennial, CO, USA). The nb identified as SEB-5 was further purified by gel filtration on an AKTA FPLC using the Superdex 200-XK 26/70 column (GE Healthcare, Marlborough, MA, USA), as previously described [48,49].

### 4.4. Production and Purification of Rabbit Polyclonal Antibodies against SEB

The polyclonal antibody (pAb) raised against the SEB toxoid was produced by Pacific Immunology Corp (Ramona, CA, USA). Briefly, the SEB toxoid (List Biological, Campbell, CA, USA) was emulsified with either complete Freund’s adjuvant (1st immunization) or incomplete adjuvant (2nd to 4th boosts) prior to immunization. The emulsion was injected into two rabbits, 14,809 and 14,810, at 3-week intervals (~300 g of toxoid was injected per rabbit at each time point). The toxoid injection did not result in cytotoxicity to either rabbit. Following the 3rd injection, bleeds were collected and evaluated for anti-SEB activity by ELISA.

Sera from the first and second bleed of rabbit 14,809 and the third and fourth bleed of rabbit 14,810 were pooled and antibodies were purified by affinity chromatography on a Protein A-conjugated agarose column (Pierce Protein A IgG Purification Kit–Thermo Scientific, Waltham, MA, USA), and then bound antibodies were eluted with 0.1 M glycine-HCl, pH 2.7. Protein concentrations were determined based on OD at 280 nm, measured with an Eppendorf BioSpectrometer (Hamburg, Germany).

### 4.5. ELISA

Sandwich ELISAs were performed as previously described [50]. Briefly, 96-well black NUNC Maxisorp flat-bottom plates (Thermo Scientific, Waltham, MA, USA) were coated with 100 μL/well of 1 μg/mL of the indicated capture antibody and incubated at 4 °C overnight. After overnight incubation, the plates were washed two times in 0.02 M Tris buffered saline with 0.9% NaCl, pH 7.4, and 0.05% Tween-20 (TBST), blocked in 5% non-fat dry milk (NFDM)-TBST or 3% BSA-TBST for an hour, and then incubated with samples in 1X PBS at room temperature for 1 h. The plates were washed in TBST six times and incubated with the indicated detection antibody (200 ng/mL in NFDM-TBST). Goat anti-mouse HRP, goat anti-rabbit HRP, goat anti-llama HRP, or streptavidin (SA)-HRP was added as a secondary antibody after washing six times in TBST. Plates were developed with 100 μL of SuperSignal West Pico Chemiluminescent Substrate (Thermo Scientific, Waltham, MA, USA) and luminescence was read on a Victor3 plate reader (Perkin Elmer, Waltham, MA, USA) over a period of 0.1 s. The limit of detection (LOD) was defined as the lowest toxin concentration at which the average ELISA reading was three standard deviations above the negative control.

A direct ELISA was performed as described above. However, plates were coated directly with 100 μL of the indicated toxin at 5 μg/mL, 100 ng/mL, 10 ng/mL, or a PBS control and incubated overnight at 4 °C. After overnight incubation, the plates were blocked in the appropriate buffer for one hour, washed twice, and then incubated with the indicated detection antibody.

### 4.6. Spiking SEB in Milk Samples

Whole milk and 2% milk were used to test for the detection of the toxin in a dairy-based product. Serial dilutions of SEB were prepared in whole and 2% milk. Spiked samples were then analyzed in a sandwich ELISA, as described above. 

### 4.7. Heat Treatment

The indicated Ab was diluted to 100 ug/mL in PBS and then incubated for ten minutes at room temperature, 63 °C, 75 °C, or 95 °C in a thermocycler. Samples were rapidly cooled by placing on ice and then diluted to 200 ng/mL for use in a direct ELISA. Plates were coated overnight with SEB at 5 μg/mL or with PBS only. Each reading was corrected by subtracting the average of the PBS control. The relative change in signal was calculated as: (corrected CPS/average of CPS at room temperature) × 100. 

### 4.8. Shelf-Life

Antibodies were stored either at room temperature or at 4 °C for 1, 8, and 16 days and used in a direct ELISA at 50 ng/mL. Plates were coated overnight with SEB at 5 μg/mL or with PBS only. Each reading was corrected by subtracting the average of the PBS control. The relative change in signal was calculated as: (corrected CPS at room temp/average corrected CPS at 4 °C) × 100.

### 4.9. Screening of nbs with Neutralization Activity

Human PBMCs were isolated from whole blood using gradient centrifugation with Lymphoprep and SepMate (StemCell Technologies). Cells were seeded at 1.5 × 10^5^ cells per well in 100 µL of R10 media in round-bottom plates. PBMCs treated with SEB (without antibodies) were used as a negative control for baseline IL-2 production. SEB (25 ng/mL) and SEB (25 ng/mL) with an equal concentration of nanobodies or pAb were co-incubated with PBMCs for 24 h. The supernatant was collected and used for a human IL-2 ELISA (Biolegend) to measure the neutralization efficacy. Results display combined data from three independent experiments, n = 9. Statistics used were the Mann–Whitney test with SEM. Here, “ns” was used to indicate *p* > 0.05 (statistically insignificant) and *, **, ***, and **** indicate statistical significance at *p* ≤ 0.05, *p* ≤ 0.01, *p* ≤ 0.001, and *p* ≤ 0.0001, respectively.

## Figures and Tables

**Table 1:** SEB nb yields and LOD ^1^ in ELISA pairing with SEB pAb.

nb	Yield (mg/L of Culture)		LOD ^1^ Using nb as a Capturer		LOD Using nb as a Detector	
	Monovalent	Bivalent	Monovalent	Bivalent	Monovalent	Bivalent
SEB-1	78	24	3.12–6.25 ^2^	0.19–0.39	3.12–6.25	0.05–0.10
SEB-5	0.32	-- ^3^	0.39	--	0.19–0.39	--
SEB-6	24	35	62.5–125	6.25	1.5–3.15	6.25
SEB-9	6.8	--	125	--	3.125	--
SEB-10	22	60	250–500	3.125	3.125–6.25	0.78
SEB-13	11	60	125–250	3.9	3.125–6.25	0.78
SEB-15	69	6.8	3.12	0.39	0.19–0.39	0.39–0.78
SEB-18	10	42	25	12.5	3.12	0.78–1.5
SEB-20	9.4	--	0.39–0.78	--	0.39	--

^1^ LOD was defined as the lowest toxin concentration at which the average ELISA reading was three standard deviations above the negative control. It is reported here in ng/mL. ^2^ Each toxin concentration in one experiment was performed in triplicate—the experiment was repeated 3 times. If the LOD obtained varied between each independent experiment, then both values are reported. ^3^ No data due to insolubility of the nb.

**Table 2:** LOD for SEB (ng/mL) from sandwich ELISA using nb pairs.

Capture	Detector	LOD
SEB-1^2^	SEB-20	0.78
SEB-5	SEB-1^2^	1.87
SEB-20	SEB-1^2^	3.75
SEB-5	SEB-9	3.75
SEB-20	SEB-9	3.75

**Table 3:** LOD for SEB-spiked (ng/mL) milk.

sdAb	Matrix	LOD
SEB-1^2^	Whole	0.19–0.39 *
	2%	0.19–0.39
	PBS	0.19–0.39
SEB-5	Whole	0.39
	2%	0.19–0.39
	PBS	0.39
SEB-15^2^	Whole	0.39
	2%	0.39
	PBS	0.39
SEB-20	Whole	0.39
	2%	0.39–0.78
	PBS	0.78

* If the LOD varied between assays, then both values are reported.

## Data Availability

Data is contained within the article or supplementary material.

## Supplementary Materials

The following supporting information can be downloaded at: https://www.mdpi.com/article/10.3390/toxins15060400/s1, Table S1: Primers used in this study. Figure S1: The activity of antisera. Figure S2: Specificity of SEB nbs.
