# Supplementary material for: Development of Thermally Stable Nanobodies for Detection and Neutralization of Staphylococcal Enterotoxin B
Source: Toxins (Basel). 2023 Jun 16;15(6):400. doi: 10.3390/toxins15060400 (PMC10301076; doi:10.3390/toxins15060400)
Supplement: Supplementary file 1 [file toxins-15-00400-s001.zip › toxins-2406358-supplementary.pdf]

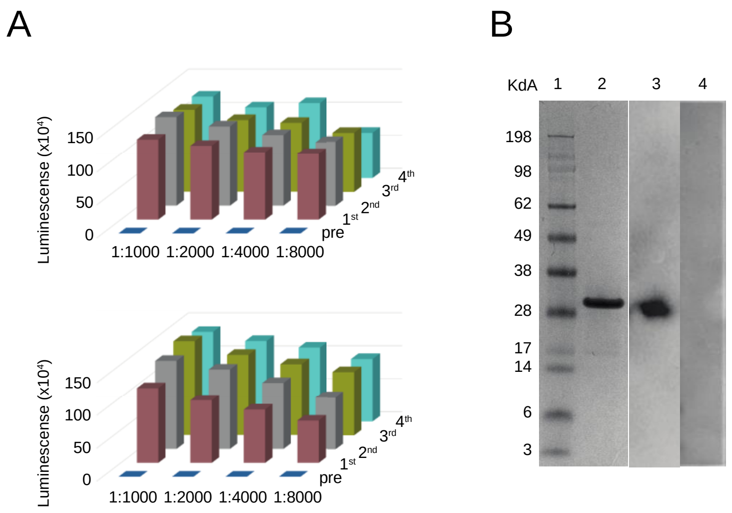

Figure S1. The activity of antisera. **A.** Data shows the binding activity of antisera from pre-immune (Pre) rabbits, 1st bleeding (one week after 3rd immunization), 2nd bleeding (two weeks after 3rd immunization), 3rd bleeding (one week after boost with additional immunogen), and 4th bleeding (three weeks after boost). ELISA was performed by coating wells with SEB (10 ng/mL), and then incubating SEB with antisera of rabbits 14809 and 14810 diluted from 1:1,000–1:8,000. Each column represents the mean of 3 replicates. The assay was repeated twice, and a representative image is shown. **B.** Reactivity of immunized rabbit serum IgG to SEB. Lane 1. Protein markers with molecular weights labeled as kilodaltons at the left side of the lane; lane 2. Coomassie stained SDS-PAGE with 1 μg of SEB; lanes 3 and 4. Western blot of 10 ng of SEB probed respectively with 0.5 μg of rabbit polyclonal antibody against SEB and rabbit pre-immune serum IgG followed by horseradish peroxidase-conjugated goat anti-rabbit IgG.

Table S1. Primers used in this study.

LIC primer F	TACTTCCAATCCAATGCA
LIC primer R	TTATCCACTTCCAATGTTATT
sdAb 5/15/6/18 F	CTGGTGGCGGAGGCAGCGGTGGCGGTGGCAGTCAGGTGCAGCTGGTGGAGT
sdAb 5 R	CGCTGCCTCCGCCACCAGATCCACCGCCTCCTGAGGAGACAGTGACCTGG
sdAb 15/20/18 R	CGCTGCCTCCGCCACCAGATCCACCGCCACCTGAGGAGACGGTGACCTGG
sdAb 20/8 F	CTGGTGGCGGAGGCAGCGGTGGCGGTGGCAGTCATGTGCAGCTGGTGGAGT
sdAB 1/8 R	CGCTGCCTCCGCCACCAGATCCACCGCCACCTGAGGAGACGGTGACCAGG
sdAB 1/13 F	CTGGTGGCGGAGGCAGCGGTGGCGGTGGCAGTGATGTGCAGCTGGTGGAGT
sdAb 6/10 R	CGCTGCCTCCGCCACCAGATCCACCGCCACCTGAGGAGACGGTGACCTGG
sdAb 10 F	CTGGTGGCGGAGGCAGCGGTGGCGGTGGCAGTGAGGTGCAGCTGGTGGAGTC

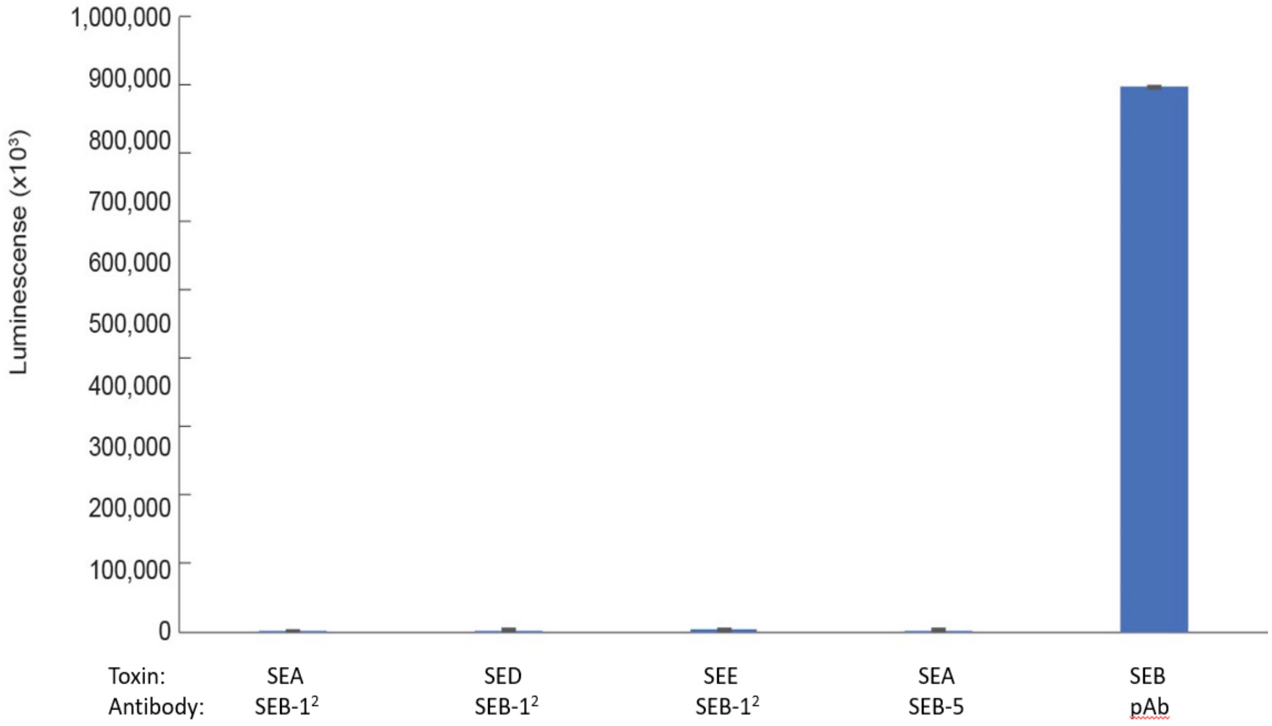

Figure S2. Specificity of SEB nbs. A representation of SEB nbs reactivity to SEA, SED, and SEE. The pAb is shown for comparison. A direct ELISA was performed by coating plates with 5 μg/mL of SE toxins, and then incubating the plates with the indicated antibodies, followed by adding appropriate secondary antibodies before signal development. ELISA signal was not corrected. Each bar represents the mean of triplicate readings ± one standard deviation from one representative experiment. Each experiment was repeated two times.
